# DsSWEET17, a Tonoplast-Localized Sugar Transporter from *Dianthus spiculifolius*, Affects Sugar Metabolism and Confers Multiple Stress Tolerance in *Arabidopsis*

**DOI:** 10.3390/ijms19061564

**Published:** 2018-05-24

**Authors:** Aimin Zhou, Hongping Ma, Shuang Feng, Shufang Gong, Jingang Wang

**Affiliations:** 1 College of Horticulture and Landscape Architecture, Northeast Agricultural University, Harbin 150030, China; aiminzhou@neau.edu.cn (A.Z.); HongpingMa@aliyun.com (H.M.); shufanggong@neau.edu.cn (S.G.); 2 Key Laboratory of Saline-Alkali Vegetation Ecology Restoration in Oil Field (SAVER), Ministry of Education, Alkali Soil Natural Environmental Science Center (ASNESC), Northeast Forestry University, Harbin 150040, China; shuangfeng1986@aliyun.com

**Keywords:** sugar transporter, tonoplast, *DsSWEET17*, *Dianthus spiculifolius*, sugar metabolism, stress tolerance

## Abstract

Plant SWEETs (Sugars Will Eventually be Exported Transporters) affect the growth of plants by regulating the transport of sugar from source to sink and its intracellular transport between different organelles. In this study, *DsSWEET17* from *Dianthus spiculifolius* was identified and characterized. Real-time quantitative PCR analysis revealed that the expression of *DsSWEET17* was affected by exogenous application of fructose and glucose as well as under salt, osmotic, and oxidation stress. Colocalization experiments showed that the DsSWEET17-GFP (green fluorescent protein) fusion protein was localized to the FM4-64-labeled tonoplasts in *Arabidopsis*. Compared to the wild type, the transgenic *Arabidopsis* seedlings overexpressing *DsSWEET17* had longer roots, greater fresh weight, and a faster root growth upon exogenous application of fructose. Furthermore, transgenic *Arabidopsis* seedlings had significantly higher fructose accumulation than was observed for the wild-type seedlings. The analysis of root length revealed that transgenic *Arabidopsis* had higher tolerance to salt, osmotic, and oxidative stresses. Taken together, our results suggest that DsSWEET17 may be a tonoplast sugar transporter, and its overexpression affects sugar metabolism and confers multiple stress tolerance in *Arabidopsis*.

## 1. Introduction

Sucrose, glucose, and fructose are the main sugars present in plants. They are the sources of carbon and energy, and their transport, distribution, and utilization play key roles in plant growth and response to biotic and abiotic stresses [1,2,3]. The translocation of sugars between the site of biosynthesis and the site of utilization or their storage depends on sugar transporters localized in different subcellular compartments and cell types [1,4]. Sugar transporters are classified into two groups, namely the H^+^-dependent and H^+^-independent transporters. Plant H^+^-independent sugar transporter SWEETs (Sugars Will Eventually be Exported Transporters) are a class of mono- and disaccharide transporters with seven conserved transmembrane domains [3,4]. They have been identified and annotated from several species with the published genomes, including *Arabidopsis thaliana* [5], *Oryza sativa* [6], and *Sorghum bicolor* [7].

SWEETs are encoded by a large family of genes, which falls into four (I, II, III, IV) phylogenetic clades [6]. In *Arabidopsis*, the AtSWEET family contains 17 members, whose roles have been gradually characterized. AtSWEET1 in clade I (AtSWEET1–3) is a glucose uniporter, localized in endoplasmic reticulum and plasma membrane [8,9]. AtSWEET4 and AtSWEET8 in clade II (AtSWEET4–8) are localized on the plasma membrane, and play an important role in the accumulation of glucose and fructose and in the cell integrity of microspores, respectively [10,11]. AtSWEET9, and AtSWEET11/12 in clade III (AtSWEET9–15) are localized on the plasma membrane, and are necessary for the secretion of nectar and sucrose phloem loading, respectively [12,13,14]. AtSWEET16 and AtSWEET17 in clade IV (AtSWEET16 and 17) are tonoplast hexose transporters and participate in the regulation of fructose levels [4,15,16,17]. These findings suggest that SWEET plays an important role in plant growth by regulating the transport of sugars. However, the functions of other members of the SWEET family have not yet been fully characterized. In addition, SWEETs from non-model wild plants is rarely identified and characterized.

*Dianthus spiculifolius* Schur, a perennial herbaceous flowering plant in the Caryophyllaceae family, exhibits strong resistance to cold and drought stress [18]. Moreover, *D. spiculifolius* exhibits a number of important application characteristics, such as a strong resistance to trampling, and a high ornamental value. In previous studies, we identified two differentially expressed genes (*DsSWEET12* and *DsSWEET17*) from transcriptome data of *D. spiculifolius* treated with cold and drought. This finding suggests that *DsSWEETs* may also be involved in plant responses to abiotic stress. Recently, we characterized the functions of *DsSWEET12* from *D. spiculifolius* [19]. DsSWEET12 is mainly localized on the plasma membrane, and may play a role in the transport and utilization of sucrose and fructose. Furthermore, overexpression of *DsSWEET12* was found to confer osmotic and oxidative stress tolerance in transgenic *Arabidopsis* plants [19]. Here, we identified DsSWEET17 as another member of the SWEET family from *D. spiculifolius*, and conducted a preliminary evaluation of its functions using transgenic *Arabidopsis* plants. The subcellular localization of DsSWEET17 was performed using green fluorescent protein (GFP) as a marker in combination with staining with a membrane marker dye, FM4-64. Furthermore, we also determined the sugar content in transgenic *Arabidopsis* and their stress tolerance. Our study should help in further characterizing the function of SWEET proteins.

## 2. Results

### 2.1. Sequence Analysis of DsSWEET17

**Figure 1:**
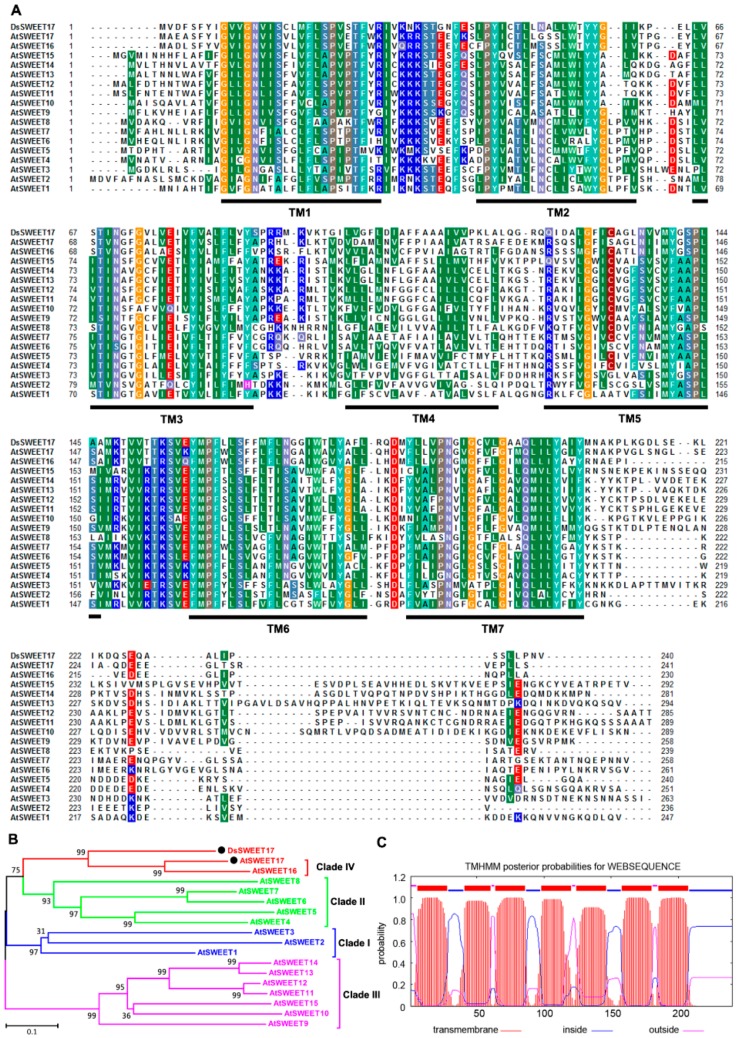
Analysis of DsSWEET17 sequence. Amino acid sequence alignment (**A**) and phylogenetic tree (**B**) of DsSWEET17 with other members (AtSWEET1 to AtSWEET17) of the AtSWEET family from *Arabidopsis*. (**A**) The same color residues indicate identical or highly similar residues in each sequence. (**C**) Putative transmembrane domains of DsSWEET17.

The open reading frame (ORF) of *DsSWEET17* was found to be 723-bp long, and was predicted to encode a protein of 240 amino acids with a molecular mass of 26.38 kDa. Multiple sequence alignment and phylogenetic analysis revealed that DsSWEET17 is most closely related to AtSWEET17 (56.38% amino acid sequence identity), belonging to clade IV of the *Arabidopsis* AtSWEET family (AtSWEET1 to AtSWEET17) (Figure 1A,B). Using the TMHMM algorithm, DsSWEET17 was predicted to have seven transmembrane regions (Figure 1A,C), which are conserved domains shared by SWEET proteins [9].

### 2.2. Expression and Subcellular Localization of DsSWEET17

**Figure 2:**
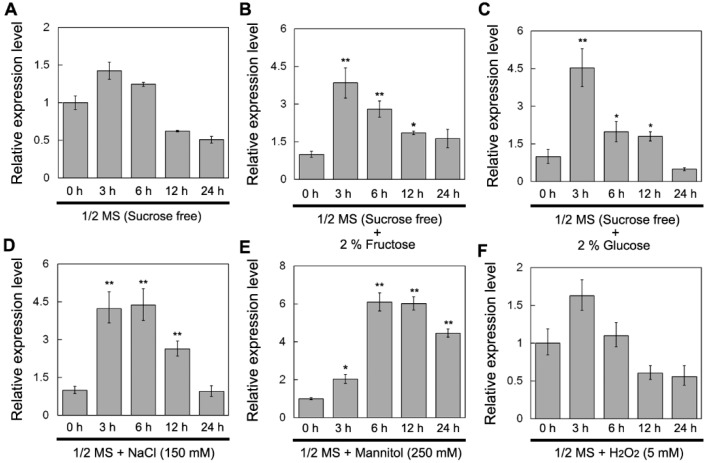
Expression analysis of *DsSWEET17* under different sugar and other stress treatments. One-week-old *D. spiculifolius* seedlings were treated with 1/2 Murashige and Skoog’s (MS) medium supplemented with sucrose (free) (**A**); fructose (2%) (**B**); or glucose (2%) (**C**); and 1/2 MS medium (3% sucrose) supplemented with NaCl (150 mM) (**D**); mannitol (300 mM) (**E**); and H_2_O_2_ (5 mM) (**F**) for 0, 3, 6, 12, and 24 h. *DsActin* was used as an internal control, and the transcript level in the untreated seedlings was set as 1.0. Asterisks indicate significant differences between untreated and stress-treated seedlings (* *p* < 0.05; ** *p* < 0.01; Student’s *t* test). Error bars show the SD of the values from three replicates.

We determined the expression of *DsSWEET17* under different sugar treatments using quantitative real-time PCR (qPCR). Under sugar free condition, the expression was slightly up-regulated at 3 h, and was then down-regulated, with the change in expression level being not more than two-fold after 24 h of treatment (Figure 2A). However, upon exogenous application of fructose (2%) or glucose (2%), the expression of *DsSWEET17* was significantly induced within 3 to 12 h of treatment, and peaked at 3 h, after which it decreased to almost the original level at 24 h (Figure 2B,C). Subsequently, we determined the changes in the expression levels of *DsSWEET17* under various abiotic stresses. Under NaCl (150 mM) and mannitol (300 mM) treatments, *DsSWEET17* expression was significantly induced within 3 to 12 h of treatment, and peaked at 6 h (Figure 2D,E). Furthermore, hydrogen peroxide (H_2_O_2_) treatment did not significantly affected the expression of *DsSWEET17*. This result suggests that the expression of *DsSWEET17* was affected by fructose and glucose as well as by multiple abiotic stresses.

**Figure 3:**
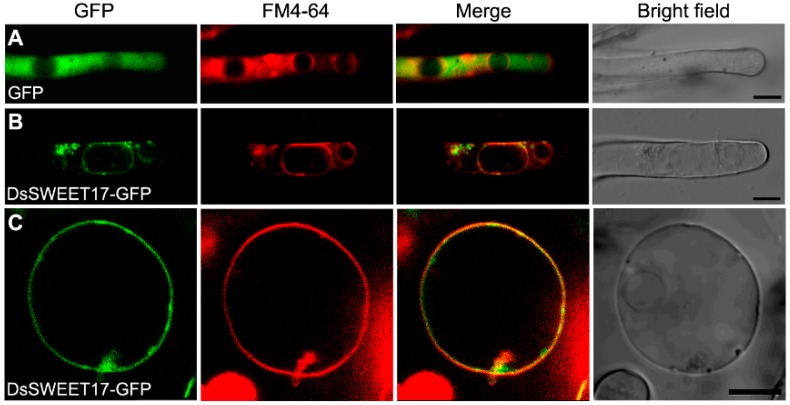
Colocalization of DsSWEET17-GFP with vacuolar membrane marker dye FM4-64 in *Arabidopsis*. *Arabidopsis* root hairs stably expressing green fluorescent protein (GFP) (**A**) or DsSWEET17-GFP (**B**) and intact vacuole isolated from root cells of transgenic *Arabidopsis* seedlings expressing *DsSWEET17-GFP* (**C**) were incubated for 30 (**A**,**B**) or 1 (**C**) min with 4 μM FM4-64. GFP fluorescence is green, and FM4-64 is red. Merge was created by merging the GFP and FM4-64 fluorescent images. Scale bars = 10 µm.

We further examined the localization of DsSWEET17 in plant cells using GFP as a fusion protein marker in combination with staining with a membrane marker dye, FM4-64. The confocal images showed that GFP was localized to the cytoplasm of root hair cells of *Arabidopsis* seedlings stably expressing GFP (Figure 3A). However, in root hair cells of *Arabidopsis* seedlings stably expressing DsSWEET17-GFP, the GFP signals were mainly observed in the membranes of vacuoles of different sizes, which were labeled by the vacuolar membrane marker dye, FM4-64 (staining was done for 30 min) (Figure 3B). Further, the intact vacuoles were isolated from root cells of transgenic *Arabidopsis* seedlings expressing *DsSWEET17-GFP*. In the intact isolated vacuole, the DsSWEET17-GFP signals were observed in the vacuolar membrane, which was also rapidly labeled by FM4-64 dye (staining was done for 1 min) (Figure 3C). This result suggests that DsSWEET17-GFP was mainly localized to the tonoplast in *Arabidopsis* cells.

### 2.3. Overexpression of DsSWEET17 in Arabidopsis Affects Seedling Growth in the Presence of Exogenous Fructose

**Figure 4:**
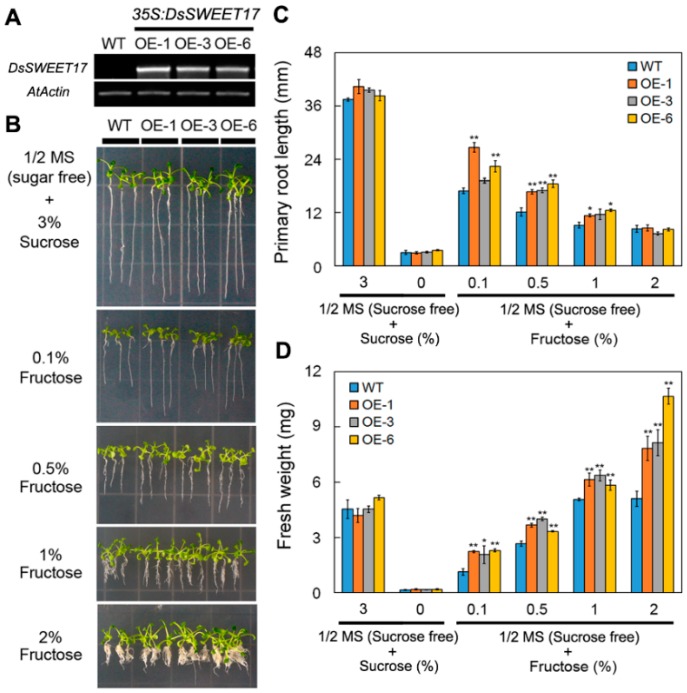
Growth of wild type (WT) and *DsSWEET17* transgenic *Arabidopsis* seedlings on different sugar (sucrose and fructose) sources. (**A**) Semi-quantitative PCR analysis of *DsSWEET17* expression in WT and transgenic *Arabidopsis* lines (OE-1, OE-3, and OE-6); (**B**–**D**) Seedling growth (**B**); root length (**C**); and fresh weight (**D**) of WT and three transgenic lines on 1/2 MS medium (sucrose free) containing different concentrations of sucrose or fructose. Asterisks indicate significant differences between WT and transgenic lines (* *p* < 0.05; ** *p* < 0.01; Student’s *t* test). Error bars show the SD of the values from three replicates.

**Figure 5:**
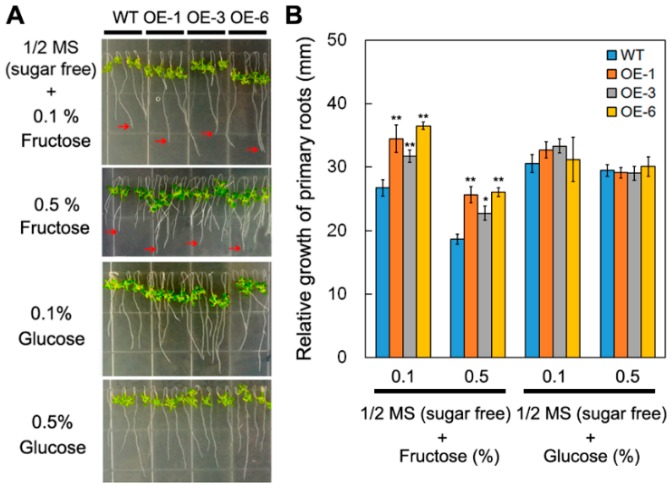
Relative root growth of wild type (WT) and *DsSWEET17* transgenic *Arabidopsis* seedlings on different sugar (fructose and glucose) sources. Seedling (**A**) and relative root growth (**B**) of WT and three transgenic lines on 1/2 MS medium (sucrose free) containing different concentrations of fructose (0.1% and 0.5%) or glucose (0.1% and 0.5%). The red arrow indicates the root tip. Asterisks indicate significant differences between WT and transgenic lines (* *p* < 0.05; ** *p* < 0.01; Student’s *t* test). Error bars show the SD of the values from three replicates.

To evaluate the role of *DsSWEET17*, transgenic *Arabidopsis* plants overexpressing *DsSWEET17* driven were generated (Figure 4A). On normal 1/2 Murashige and Skoog’s (MS) medium (3% sucrose) or 1/2 MS medium without sucrose, there was no significant difference in the growth of transgenic and wild-type (WT) seedlings as assessed by measuring the root length and fresh weight. However, on 1/2 MS medium (sucrose free) supplemented with various concentrations of exogenous fructose, the root length (at 0.1% and 0.5% concentrations) and fresh weight (at 0.1%, 0.5%, 1%, and 2% concentrations) of transgenic seedlings were significantly higher than those of WT seedlings (Figure 4B–D). Furthermore, the analysis of relative root growth showed that the roots of the transgenic seedlings grew faster than those of WT seedlings on 1/2 MS medium (sucrose free) containing 0.1% or 0.5% fructose, but not on the medium containing 0.1% or 0.5% glucose (Figure 5A,B). These results suggest that the overexpression of *DsSWEET17* in *Arabidopsis* affects the seedling growth in a fructose-dependent manner.

### 2.4. Overexpression of DsSWEET17 in Arabidopsis Affects Sugar Metabolism and Confers Tolerance to Multiple Stresses

**Figure 6:**
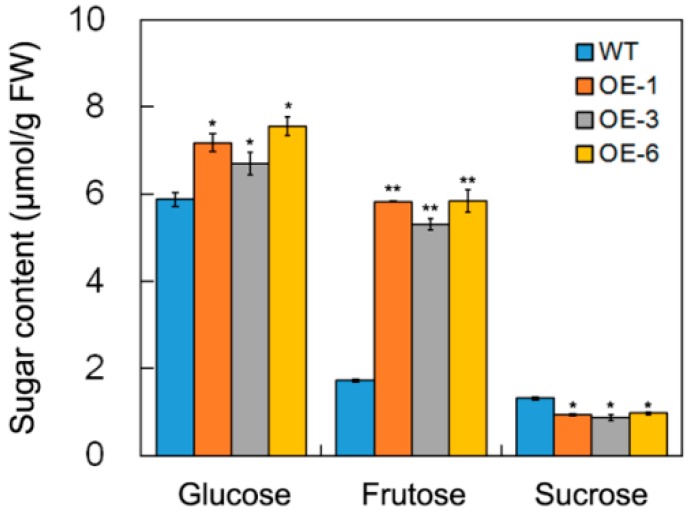
Sugar content in wild type (WT) and *DsSWEET17* transgenic *Arabidopsis* (OE-1, OE-3, and OE-6) seedlings. The sucrose, fructose, and glucose contents were measured in seedlings grown on 1/2 Murashige and Skoog’s (MS) medium (3% sucrose) for 3 weeks. Asterisks indicate significant differences between WT and transgenic lines (* *p* < 0.05; ** *p* < 0.01; Student’s *t* test). Error bars show SD of the values from three replicates.

Sugar content in transgenic and WT seedlings were measured. We observed that in the WT seedlings, the contents of glucose and fructose in the transgenic seedlings were increased, whereas that of sucrose was reduced (Figure 6). Of the three sugars, the change in fructose content was the most significant. This result suggests that the overexpression of *DsSWEET17* in *Arabidopsis* affects the metabolism of sugars, especially that of fructose.

**Figure 7:**
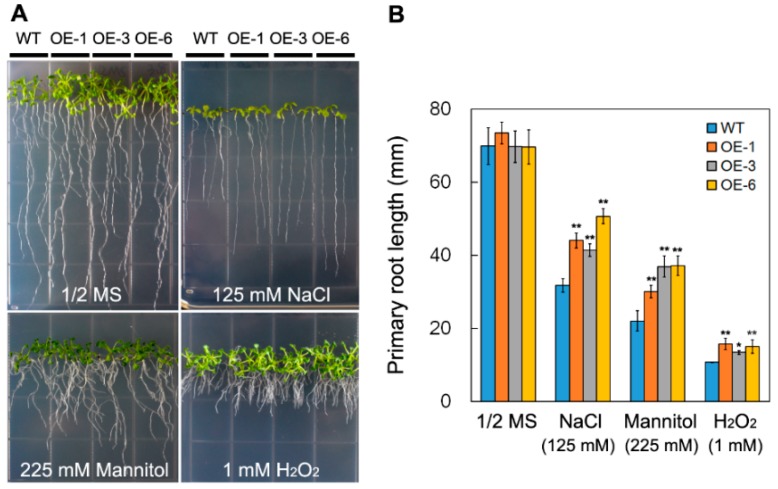
Phenotypes of wild type (WT) and *DsSWEET17* transgenic *Arabidopsis* seedlings under various stresses. Seedling growth (**A**); and root length (**B**) of WT and three transgenic lines on 1/2 Murashige and Skoog’s (MS) medium or 1/2 MS medium supplemented with NaCl (125 mM), mannitol (225 mM), and H_2_O_2_ (1 mM). Asterisks indicate significant differences between WT and transgenic lines (* *p* < 0.05; ** *p* < 0.01; Student’s *t* test). Error bars show the SD of the values from three replicates.

The expression of *DsSWEET17* was induced by salt, osmotic, and oxidation stresses. Therefore, we compared the phenotypes of the transgenic and WT seedlings under salt, osmotic, and oxidative stress. On 1/2 MS medium, there was no significant difference in growth between the transgenic and wild-type (WT) seedlings as assessed by measuring the root length. However, on 1/2 MS media supplemented with NaCl (125 mM), mannitol (225 mM), or H_2_O_2_ (1 mM), the length of roots of the transgenic seedlings was significantly greater than those of WT plants (Figure 7A,B). This result suggests that the overexpression of *DsSWEET17* in *Arabidopsis* confers tolerance to salt, osmotic, and oxidative stresses.

## 3. Discussion

In *Arabidopsis*, AtSWEET17 is a tonoplast fructose transporter that controls the content of fructose in leaves [16,17]. In our study, qPCR analysis showed that the expression of *DsSWEET17* was induced by fructose (Figure 2B). Colocalization experiments confirmed that DsSWEET17-GFP was mainly localized to the tonoplast, which was labeled with the vacuolar membrane marker, FM4-64, dye, in *Arabidopsis* root cells (Figure 3B,C). In the absence of sucrose, exogenous application of fructose promoted the growth of transgenic *Arabidopsis* seedlings as indicated by longer roots, larger fresh weight, and faster root growth, compared to the respective parameters in the wild type seedlings (Figure 4 and Figure 5). Furthermore, the analysis of sugar content showed that the content of fructose in the transgenic seedlings was significantly higher than that in the WT seedlings (Figure 6), indicating that the overexpression of *DsSWEET17* affects the accumulation of fructose in *Arabidopsis*. In addition, the exogenous application of glucose also induced the expression of *DsSWEET17*, but did not affect the growth of transgenic *Arabidopsis* seedlings expressing *DsSWEET17*. We speculated that the exogenous application of glucose might result in changes in the expression of *DsSWEET17* by affecting the fructose content, because sucrose, fructose and glucose contents are in dynamic balance in plant cells. The analysis of sugar content may provide evidence for the validation of this speculation. In addition to the significant increase in fructose accumulation, the glucose content in transgenic *Arabidopsis* seedlings expressing *DsSWEET17* was also increased, whereas the sucrose content was decreased, compared to the respective contents in the wild type seedlings. This result suggests that the overexpression of *DsSWEET17* affected the sugar metabolism by affecting the accumulation of fructose in the *Arabidopsis* seedlings. We speculated that the change in fructose content might be caused by the expression of *DsSWEET17* driven by the 35S promoter in the entire plant. The overexpression of *DsSWEET17* may affect the fructose transport and might indirectly affect the sugar metabolism. Sugar is necessary for plant growth and development as carbon and energy sources [20]. Taken together, our results suggest that DsSWEET17 is a tonoplast sugar transporter, and its overexpression affects the seedling growth and sugar metabolism by changing the transport or utilization of fructose in *Arabidopsis*. Similarly, previous reports have shown the important role of AtSWEET17 in the transport and utilization of fructose [16].

To date, the function of SWEET17 in the response of plants to abiotic stress is poorly studied. The qPCR analysis showed that the expression of *DsSWEET17* was also induced by NaCl and mannitol treatment (Figure 2D,E). The analysis of root length showed that transgenic *Arabidopsis* had higher tolerance to salt (125 mM NaCl), osmotic (225 mM mannitol), and oxidative (1 mM H_2_O_2_) stresses (Figure 7), indicating that the overexpression of *DsSWEET17* also improved the tolerance of *Arabidopsis* to salt, osmotic, and oxidative stresses. Sugar content analysis showed that glucose and fructose contents in transgenic *Arabidopsis* seedlings were significantly higher than those in wild type (Figure 6). Therefore, we speculated that higher sugar accumulation may be closely related to stronger tolerance of salt and osmotic stress in transgenic *Arabidopsis*. Because sugar, as an osmotic regulator, is involved in the regulation of osmotic balance in plant cells under salt and drought stress [1]. Studies shown that drought induced accumulation of soluble sugar [21]. In addition, studies have shown that sugar serves as a signal involved in the reactive oxygen species (ROS) scavenging pathway under oxidative stress [22].

In summary, our results demonstrate that the overexpression of *DsSWEET17* confers tolerance to salt, osmotic, and oxidative stresses by affecting the sugar metabolism in *Arabidopsis*.

## 4. Materials and Methods

### 4.1. Identification of DsSWEET17 and Sequence Analysis

The *DsSWEET17* gene was identified from the transcriptome sequencing data of *D. spiculifolius* [18]. Amino acid sequence alignment and transmembrane domains prediction of DsSWEET17 were performed using ClustalW (available online: http://www.clustal.org/clustal2/) and TMHMM (available online: http://www.cbs.dtu.dk/services/TMHMM/) algorithms, respectively. The phylogenetic tree was constructed using molecular evolutionary genetics analysis (MEGA) 4.1 software (available online: http://www.megasoftware.net/).

### 4.2. Plant Material and Growth Conditions

All *Arabidopsis* plants used in this study belonged to the Columbia-0 ecotype. The *Arabidopsis* seeds were surface sterilized and treated at 4 °C for 2 days, then grown on 1/2 strength MS medium (3% sucrose, 1% agar, pH 5.8) under a 12 h light/12 h dark photoperiod (100 μmol·m^−2^·s^−1^ light intensity) at 22 °C.

The *D. spiculifolius* seeds were surface sterilized and germinated on 1/2 MS medium (3% sucrose). For stress treatments, 1-week-old *D. spiculifolius* seedlings were exposed to 1/2 MS medium (sucrose free), and 1/2 MS medium (sucrose free) supplemented with 2% fructose or 2% glucose, and 1/2 MS medium (3% sucrose), supplemented with 150 mM NaCl, 300 mM mannitol, or 5 mM H_2_O_2_. Seedlings were harvested at different time points (0, 3, 6, 12, or 24 h), frozen immediately in liquid nitrogen for RNA preparation.

### 4.3. RNA Extraction and Quantitative Real-Time PCR Analyses

Total RNA was extracted using an RNeasy^®^ Mini Kit (Qiagen, Valencia, CA, USA). First-strand cDNA was synthesized using an M-MLV RTase cDNA Synthesis Kit (TaKaRa, Shiga, Japan). Real-time quantitative PCR (qPCR) analysis was performed using SYBR^®^ Green Mix (Agilent Technologies, Palo Alto, CA, USA) on an Mx3000P system (Agilent Technologies). Three biological replicates and three technical replicates were performed for each analysis. The primers used in this study are shown in Table 1.

### 4.4. Vector Construction and Plant Transformation

For the vector construction, the open reading frame (ORF) of *DsSWEET17* was amplified by PCR and cloned at the BamHI and SacI sites of the pBI121 vector. To construct the GFP fusion genes, the ORF of *DsSWEET17*, without the stop codon, was amplified by PCR and cloned at the BamHI and AgeI sites of the pBI121-GFP vector. These constructs were transformed into *Arabidopsis* by *Agrobacterium tumefaciens* (EHA105)-mediated floral dip method [23]. The transgenic *Arabidopsis* lines were evaluated by semi-quantitative reverse transcription PCR. The primers used in this study are shown in Table 1.

### 4.5. Confocal Laser Scanning Microscopy and FM4-64 Staining

The isolation of intact vacuoles, and FM4-64 staining of seedling roots and isolated vacuoles was performed as described previously [24]. Roots of *Arabidopsis* seedlings (5-day-old) with stable expression of DsSWEET17-GFP were incubated in 1 mL of liquid 1/2 MS medium (0.5% (*w*/*v*) sucrose, pH 5.8) containing 4 µM FM4-64 (Invitrogen, Carlsbad, CA, USA) for 30 min. Roots were washed twice with liquid 1/2 MS medium shortly before confocal laser scanning microscopy (CLSM) (Nikon, A1, Tokyo, Japan). The GFP (500–530 nm emission filter) and FM4-64 (620–680 nm emission filter) signals were visualized using confocal laser scanning microscopy (CLSM; Nikon, A1, Tokyo, Japan).

For the isolation of intact vacuoles from *Arabidopsis* root cells expressing DsSWEET17-GFP, root tips (2–3 cm) were chopped into 0.5-mm-long fragments and incubated in the enzyme solution (1% cellulase Onozuka R10, 0.25% macerozyme R10, 0.4 M mannitol, 10 mM CaCl_2_, 20 mM KCl, 0.1% BSA, and 20 mM MES, pH 5.7) at 28 °C with gentle shaking at 60 rpm for 6 h. Isolated vacuoles were incubated in W5 solution (154 mM NaCl, 125 mM CaCl_2_, 5 mM KCl, 5 mM glucose, and 2 mM MES, pH 5.7) that contained 4 µM FM4-64 for 1 min and then immediately observed using CLSM.

### 4.6. Analysis of the Sugar Content

*Arabidopsis* seedlings grown on 1/2 MS medium (3% sucrose) for 3 weeks were used to measure the sugar content. To measure sugars, *Arabidopsis* seedlings (0.2 g fresh weight, FW) were homogenized in 2 mL of hyperpure water, centrifuged at 8000× *g* for 10 min at 4 °C. After passing through a 0.22 mm filter, a 10 μL sample was injected into a Kro-masil^®^ NH2 column (4.6 × 250 mm; (AkzoNobel, Bohus, Sweden), and sugar contents were analyzed by high performance liquid chromatography (HPLC) (Waters 510, Waters Associates Inc., Milford, MA, USA). The experiment was replicated three times.

### 4.7. Sugar Treatment and Stress Tolerance Assay

For sugar and stress treatment tests, *Arabidopsis* seeds were surface sterilized and treated at 4 °C for 2 days. Subsequently, seeds were grown vertically for 14 days on 1/2 MS medium (sucrose free) containing different concentrations of sucrose (3% and 0%), fructose (2%, 1%, 0.5%, and 0.1%), and glucose (0.5%, and 0.1%) or 1/2 MS medium (3% sucrose) supplemented with NaCl (125 mM), mannitol (225 mM), and H_2_O_2_ (1 mM) before root length and fresh weight measurements were taken. The experiment was replicated three times.

For root relative growth tests, the seedlings were germinated on 1/2 MS medium (sucrose free) for 5 days, and were then transplanted to 1/2 MS medium (sucrose free) containing different concentrations of fructose (0.5% and 0.1%) and glucose (0.5% and 0.1%) for 7 days (inverted culture). The experiment was replicated three times.

## Figures and Tables

**Table 1:** List of primers used in this study.

Primer Name	Primer Sequence (5′→3′)	Purpose
DsSWEET17-qF	CATCAATGGTTTCGGTGTTCT	qPCR
DsSWEET17-qR	TGACGCCCTTGTAACGCTAA	qPCR
DsActin-qF	CGGTGGCTCTATCCTCGCTT	qPCR
DsActin-qR	TTCCTGTGGACGATTGACGG	qPCR
AtActin1-F (AT2G37620)	GAAAATGGCTGATGGTGAAG	RT-PCR
AtActin1-R	CTCATAGATAGGAACAGTGTGGC	RT-PCR
DsSWEET17 (BamHI)-F	GGATCCATGGTGGATTTTAGCTTCT	Cloning and Subcellular localization
DsSWEET17 (SacI)-R	GAGCTCTTAAACATTTGGAAGTAGAC	Cloning
DsSWEET17 (AgeI)-R	ACCGGTACATTTGGAAGTAGACTAGAAG	Subcellular localization
